# Using optimized CT type to predict histological classifications of thymic epithelial tumors: a radiomics integrated analysis

**DOI:** 10.1186/s13244-025-01933-7

**Published:** 2025-03-22

**Authors:** Zhengping Zhang, Kede Mi, Zhaojun Wang, Xiaoyan Yang, Shuping Meng, Xingcang Tian, Yanzhu Han, Yuling Qu, Li Zhu, Juan Chen

**Affiliations:** 1 Department of Key Laboratory of Ningxia Stem Cell and Regenerative Medicine, Institute of Medical Sciences, General Hospital of Ningxia Medical University, Yinchuan, China; 2 Department of Radiology, General Hospital of Ningxia Medical University, Yinchuan, China; 3 Department of Medical, General Hospital of Ningxia Medical University, Yinchuan, China; 4 Department of Critical Care Medicine, General Hospital of Ningxia Medical University, Yinchuan, China; 5 Department of Pulmonary and Critical Care Medicine, General Hospital of Ningxia Medical University, Yinchuan, China; 6 Department of Pathology, General Hospital of Ningxia Medical University, Yinchuan, China

**Keywords:** Computed tomography, Thymic epithelial tumors, Radiomics

## Abstract

**Objective:**

To develop and externally validate an integrated model that utilizes optimized radiomics features from non-contrast-enhanced CT (NE-CT) or contrast-enhanced CT (CE-CT), along with morphological features and clinical risk factors, to predict histological classifications of thymic epithelial tumors (TETs).

**Methods:**

A total of 182 patients with TET, classified as the low-risk group and the high-risk group based on histology, were divided into a training cohort (*N* = 122, center 1) and an external validation cohort (*N* = 60, center 2). Radiomics features were extracted from different CT types, followed by feature selection, including consistency, correlation, and importance tests, to generate Rad-scores for both NE-CT and CE-CT. The integrated model was developed by combining the optimal Rad-score, morphological features, and clinical risk factors using multivariate logistic regression. Model performance was assessed by the area under the receiver operating characteristic curve (AUC) and compared by Delong test. A nomogram was used to visually present the integrated model.

**Results:**

A total of 851 radiomics features were extracted, with NE-CT and CE-CT Rad-scores consisting of four and five features, respectively. The AUCs of the CE-CT Rad-score were higher than those of the NE-CT Rad-score in both the training cohort (0.783 vs 0.749) and the external validation cohort (0.775 vs 0.723, *p* = 0.361). The integrated model, combining five morphological features and the CE-CT Rad-score, achieved AUCs of 0.814 and 0.802 in the training and external validation cohorts, respectively.

**Conclusion:**

The integrated model, incorporating radiomics features from CE-CT and morphological features, can help to identify the histological classifications of TETs.

**Critical relevance statement:**

This study developed an integrated model based on radiomics features from contrast-enhanced CT and morphological features, demonstrating that the integrated model has impressive predictive capability in distinguishing histological classifications of thymic epithelial tumors through external validation.

**Key Points:**

- Radiomics features extracted from CT more effectively represented thymic epithelial tumor (TET) heterogeneity than morphological features.
- The radiomics model using contrast-enhanced CT outperformed that using non-contrast-enhanced CT in identifying histological classifications of TET.
- The integrated model, combining radiomics and morphological features, exhibited the highest performance in predicting TET histological classifications.

**Graphical Abstract:**

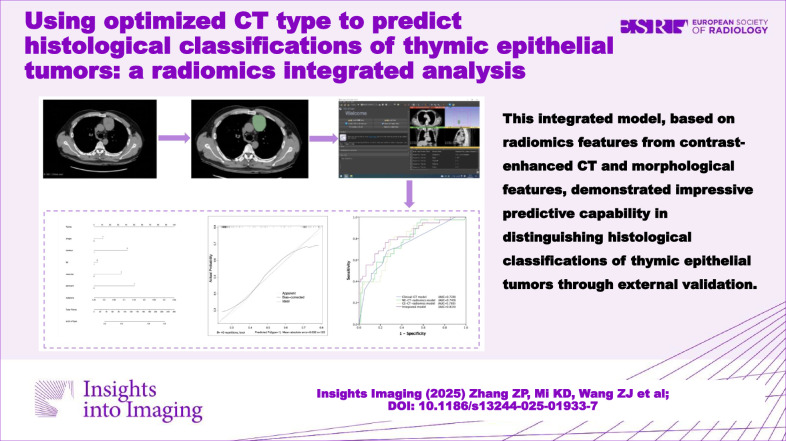

## Introduction

Thymic epithelial tumor (TET) constitutes 47% of anterior mediastinal tumors, with 30–60% of thymoma cases demonstrating invasion of the capsule and surrounding tissues and organs [1]. The histological classifications of TETs are categorized into low-risk and high-risk groups according to the invasiveness and prognostic variations among pathological subtypes [2]. In contrast to the low-risk group, the high-risk group demonstrates a poorer prognosis and a higher probability of metastasis and recurrence [2, 3], but the utilization of preoperative neoadjuvant chemotherapy and radiotherapy is beneficial in terms of tumor progression and improving overall survival rate. Therefore, accurate prediction of histological classifications of TETs before surgery is crucial for prognosis evaluation and treatment selection [4, 5]. The needle biopsy is a reliable diagnostic method for TETs; however, a small biopsy specimen may not always accurately represent the entire tumor. Additionally, deep biopsy, an invasive procedure, carries the risk of complications [6, 7].

Chest CT is the most commonly used imaging modality for the preoperative evaluation of TETs. Some studies have shown that CT can preliminarily determine the histological classifications of tumors through conventional imaging features of lesions [8]. However, these features are often insufficient to fully and reliably reflect the subtypes of TETs, primarily due to their subjectivity and limited information. Consequently, there is an urgent need for a more effective and objective approach to preoperatively determine the subtype of TETs.

Radiomics is a method that converts visual image information into profound-level characteristics, facilitating quantitative analysis [9]. The quantitative features derived from radiomics offer insights into tumor phenotype and micro-environment, revealing tumor heterogeneity [10, 11]. Several studies have demonstrated a correlation between radiomics features derived from contrast-enhanced CT (CE-CT) or non-contrast-enhanced CT (NE-CT) and the histological classifications of TETs [12–16]. However, few studies have compared the performance of radiomics models between CE-CT and NE-CT. Therefore, the aim of this study is to identify the optimal radiomics features from CE-CT and NE-CT for predicting TET histological classifications. Additionally, an integrated model is developed by combining optimized radiomics features, morphological features, and clinical risk factors to differentiate the histological classifications of TETs.

## Materials and methods

This study was approved by the Ethics Committee of our hospital (KYLL-2021-632) and conducted following the Declaration of Helsinki.

### Study protocol and patients

**Fig. 1:**
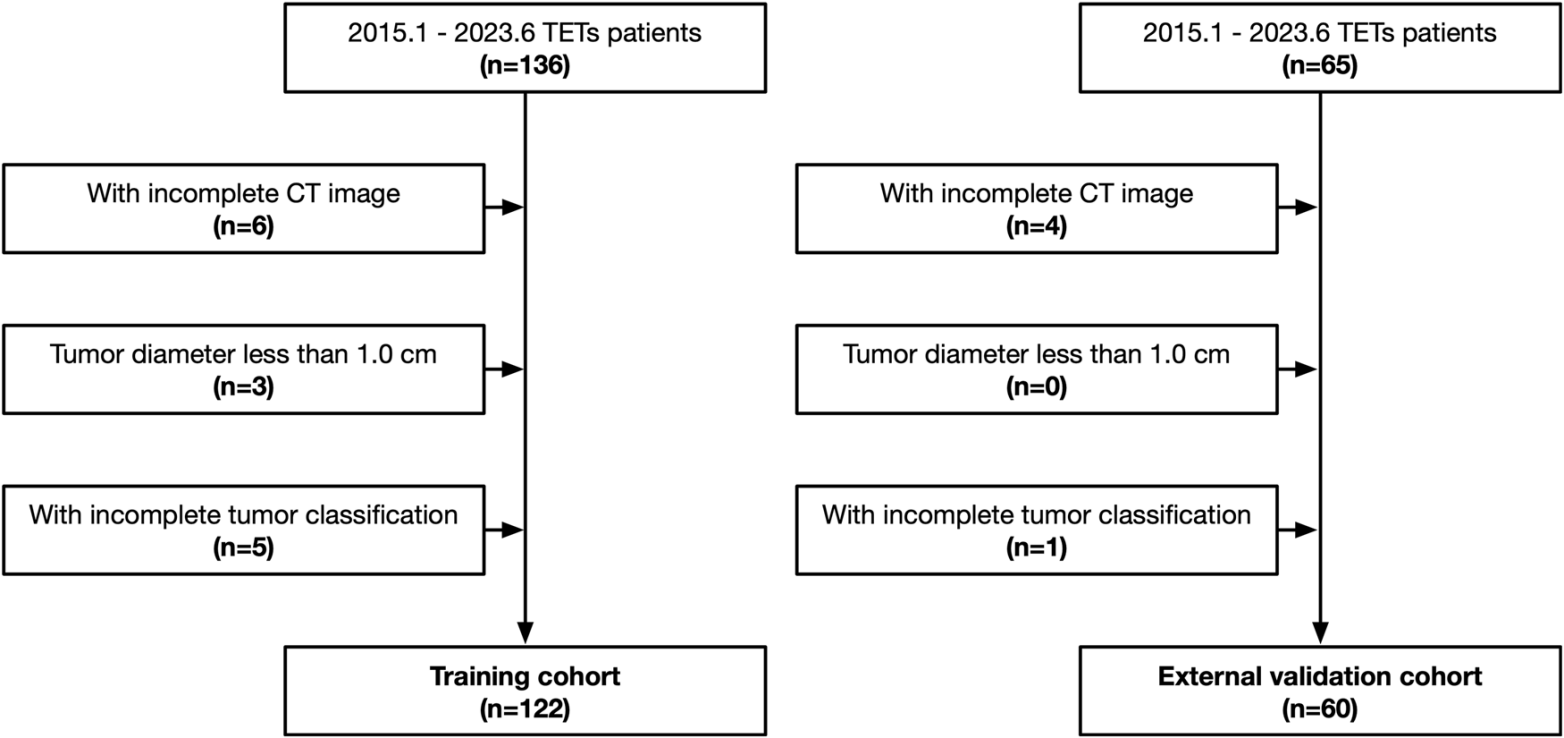
The flowchart of inclusion and exclusion criteria for patients with thymic epithelial tumors (TETs)

A total of 182 patients with TETs confirmed by surgical pathology were retrospectively enrolled from the General Hospital of Ningxia Medical University (training cohort: 122) and the General Hospital of Ningxia Medical University Hospital of Cardio-Cerebral-Vascular-Disease (external validation cohort: 60) between January 2015 and December 2023 (Fig. 1). Tumor histological classification follows the 2015 WHO Classification of Thymic Epithelial Tumors [2]. Exclusion criteria included: (1) patients with incomplete or low-quality CT image data; (2) tumor diameter less than 1.0 cm; (3) incomplete tumor classification and immunohistochemical information; (4) prior radiotherapy or chemotherapy before CT examination.

### Image acquisition

CT imaging was performed before treatment using the CT scanner (German SOMATOM dual-source 64 detector row, revolution 128 detector row, General Electronics) for the training cohort, and the CT scanners (Gem 64 detector row, General Electronics) for the external validation cohort. Specifically, 65, 57, and 60 cases were scanned using the SOMATOM (dual-source 64 detector row), GE revolution (128 detector row), and GE Gem (spectrum 64 detector row) CT scanners, respectively. Scanning protocols were consistent across center, with patients in a supine position and the scanning range from the lung apex to base. Scanning parameters for both NE-CT and CE-CT were as follows: the tube voltage of CT = 120 kV, tube current = 120–200 mAs, layer thickness = 5 mm, layer spacing = 5 mm, spiral pitch = 0.980, the reconstructed layer thickness and layer spacing = 1.25 mm, FOV = 35 cm, and matrix size = 512 × 512. Iopromide was employed as the contrast agent (the concentration of iodine was 300 mg/mL), with a flow rate of 3–4 mL/s and a dose of 1.0 mL/kg. Iopromide was administered through the anterior elbow vein via a high-pressure syringe, and the monitoring trigger threshold for the thoracic aorta was 80 Hu. A scanning was performed in the pulse phase, and the scanning in the venous phase was performed at 45 s.

### Pathology

Lesions were classified by senior pathologists using a blind method into Type A, Type AB, Type B1, Type B2, Type B3, and thymic carcinoma, according to the 2015 WHO Histological Classifications of Thymic Epithelial Tumors [2]. For analysis, these classifications were grouped into a low-risk group (A, AB, B1) and a high-risk group (B2, B3, thymic carcinoma).

### Tumor image segmentation

**Fig. 2:**
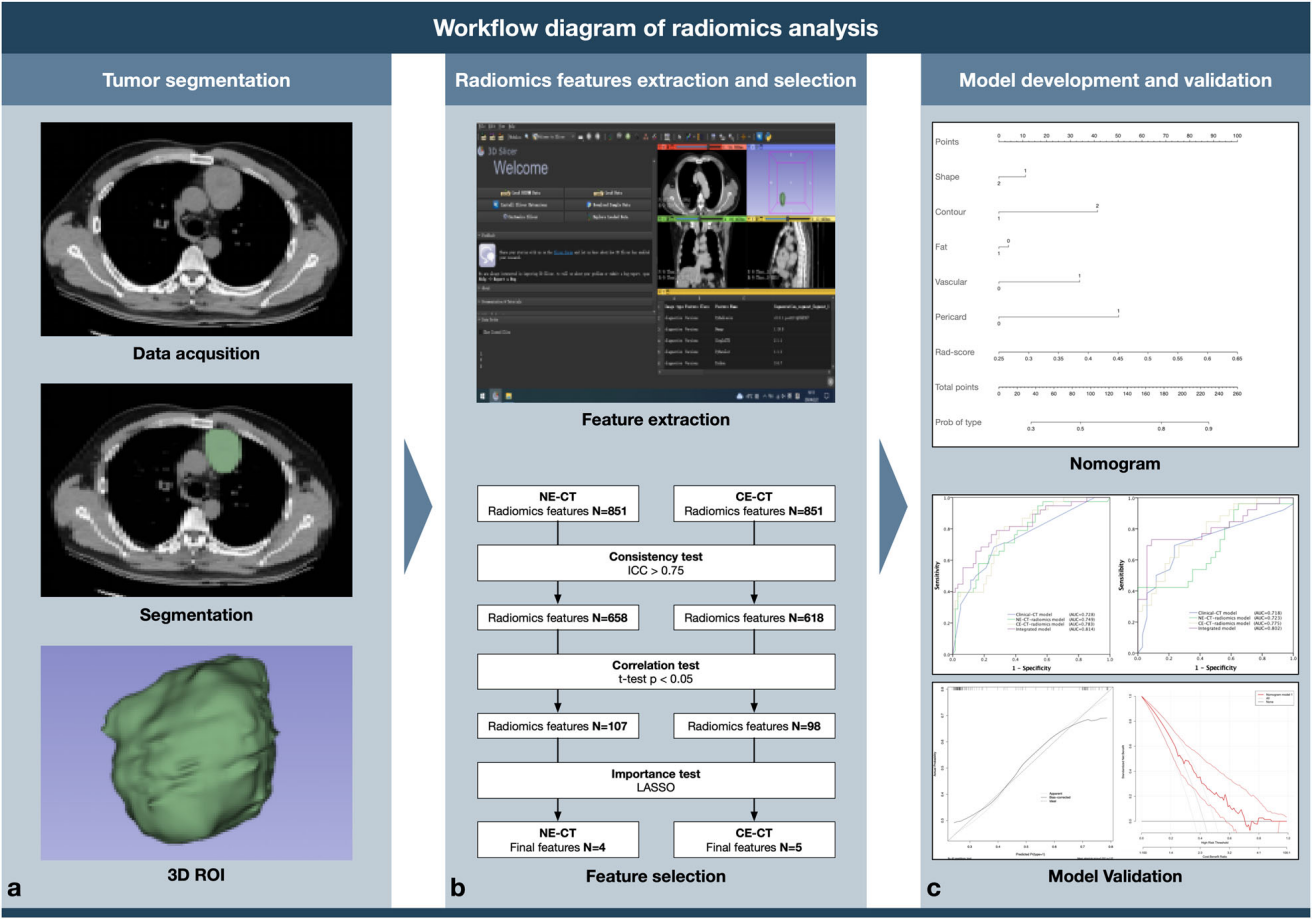
Workflow diagram of the study. **a** The workflow for tumor region segmentation; **b** the workflow for radiomics features extraction and selection; and (**c**) the methods of model development and evaluation

The 3D Slicer software (Version 5.2.1, https://www.slicer.org) was used to manually segment the tumor region of interest on NE-CT and CE-CT images by two radiologists (XCT and SPM, each with over 10 years of experience in chest imaging) using a blind method (Fig. 2a). The venous phase was selected for the enhanced sequence, as contrast agent artifacts in the arterial phase of the superior vena cava significantly affect tumor imaging. Tumor contours were delineated on each slice to generate a complete 3D volume of interest (VOI) for the tumor. The delineation margin remained within 1–2 mm of the tumor edge to prevent interference from surrounding fat structures, and adjacent blood vessels and cardiac structures were avoided.

### Radiomics features extraction and selection

As shown in Fig. 2b, for both NE-CT and CE-CT, radiomics features were extracted, and feature selection was performed separately. Radiomics features were extracted from the VOI on CT images using PyRadiomics (Version 2.1.0) within 3D Slicer. All images were re-sampled to the same voxel size (2 × 2 × 2 mm) to standardize the volume. A total of 851 radiomics features were extracted, including 18 first-order statistical features, 14 shape features, 14 gray level dependence matrix (GLDM) features, 16 gray level size zone matrix (GLSZM) features, 24 gray level co-occurrence matrix (GLCM) features, 16 gray level run length matrix (GLRLM) features, 5 neighboring graytone difference matrix (NGTDM) features, and 744 wavelet features derived from the wavelet transformation of raw images. Before feature selection, maximum-minimum standardization was applied to each radiomics feature.

Feature selection included three steps: consistency test, correlation test, and importance test. To ensure stability among observers, an initial consistency test was conducted on radiomics features. The intraclass correlation coefficient (ICC) was used to assess features consistency related to lesion delineation. For inter-observer consistency, 30 patients were randomly selected and independently delineated by the mentioned two radiologists (XCT and SPM), and the consistency of their radiomics features was evaluated using ICC. For intra-observer consistency, the same 30 patients were re-delineated by radiologist XCT, and ICC was used to assess consistency between the two delineations by the same radiologist. An ICC above 0.75 indicated satisfactory consistency, and features with ICCs equal to or below 0.75 were excluded [17]. Next, a correlation test was conducted using a *t*-test to identify features correlated with TET histological classifications. Features with *t*-test *p*-value of 0.05 or higher were excluded.

Lastly, an importance test based on the least absolute shrinkage and selection operator (LASSO) regression method was applied to select the most relevant radiomics features. LASSO regression was performed on the training cohort to identify features with minimal redundancy and maximum robustness, enhancing classification separability and representativeness of lesions. The complexity of LASSO regression was adjusted using the λ parameter, with increasing λ imposing a greater penalty on each feature’s coefficient, thereby reducing the number of non-zero coefficients. Significant radiomics features with non-zero coefficients were selected based on results from cross-validation LASSO regression with a fixed random seed, generating a radiomics score (Rad-score) for each patient. The Rad-score was calculated by multiplying each significant radiomics feature by its corresponding coefficient and summing the results.

### Clinical risk factors and conventional CT imaging signs

Clinical risk factors included age, gender, and myasthenia gravis. Tumor morphological features, including size, shape, contour, density, calcification, fatty infiltration, macrovascular, and pericardium invasion, were independently elevated by two radiologists. The shape was defined by the ratio of the longest to the shortest diameter of the tumor; round: ratio less than 3.0; irregular: ratio greater than or equal to 3.0. Contour: smooth, not smooth. Size: the axial image was measured at the level with the largest tumor diameter. Density: the difference in CT values between adjacent areas within the lesion in the mediastinal window less than 10 HU was considered uniform density, and vice versa. Cystic changes, necrosis, and calcification were also evaluated. Adjacent tissue involvement: fat, large blood vessels, and pericardium.

### Model development and evaluation

Multivariate logistic regression was used to develop models for distinguishing the low-risk and high-risk groups based on different combinations of features. A clinical-CT model was developed using clinical risk factors and morphological features, while The NE-CT and CE-CT radiomics models corresponded to the NE-CT and CE-CT Rad-scores, respectively. An integrated model was developed that included clinical risk factors, morphological features, and the optimal Rad-score.

Model discrimination performance was assessed using receiver operating characteristic (ROC) and area under the receiver operating characteristic curve (AUC). Binary variables with AUCs close to 1 indicated higher discrimination levels. An AUC less than 0.6 denoted a low discrimination level, an AUC between 0.6 and 0.75 denoted a medium discrimination level, and an AUC greater than 0.75 denoted a high discrimination level. The calibration performance and clinical applicability of the integrated model were further evaluated using calibration curves and decision curve analysis (DCA) in the external validation cohort. A nomogram was constructed to visually represent the integrated model (Fig. 2c).

### Statistical analysis

Measurement data were represented by mean ± standard deviation, while categorical data were represented as rates. The Mann–Whitney U test and chi-square test were used to compare continuous variables and categorical variables, respectively, between low-risk and high-risk groups. The Delong test was used for statistical comparisons of AUCs. Bootstrapping (*n* = 1000) was conducted to calculate the 95% confidence interval (CI). Data analysis was performed using R software (Version 4.2.2) and SPSS (version 26.0). A two-tailed *p* < 0.05 denoted statistical significance.

## Results

### Clinical and CT parameters

As shown in Table 1, age, gender, and myasthenia gravis did not differ significantly between the low-risk and high-risk TET groups in either cohort (*p* > 0.05). In the training cohort, there were 68 low-risk patients (Type A: 13, Type AB: 31, Type B1: 24) and 54 high-risk patients (Type B2: 27, Type B3: 13, thymic carcinoma: 14). In the external validation cohort, there were 34 low-risk patients (Type A: 4, Type AB: 15, Type B1: 15) and 26 high-risk patients (Type B2: 13, Type B3: 7, thymic carcinoma: 6). For morphological features, significant differences were observed in shape, contour, fatty infiltration, and macrovascular and pericardium invasion of tumors between low-risk and high-risk groups in both cohorts (*p* < 0.05).

**Table 1:** Clinical risk factors and morphological features between low-risk and high-risk groups

	Training cohort (*n* = 122)			External validation cohort (*n* = 60)		
	Low risk (68)	High risk (54)	*p*-value	Low risk (34)	High risk (26)	*p*-value
Gender (M/F)	35/33	22/32	0.205	14/20	15/11	0.313
Age (years)	50 ± 16	46 ± 17	0.163	47 ± 16	44 ± 17	0.399
Gravity (*n*, %)	9 (13.24)	8 (14.81)	0.802	5 (14.71)	2 (7.69)	0.454
Histological classifications (*n*, %)						
Type A	13 (19)	\	\	4 (12)	\	\
Type AB	31 (46)	\	\	15 (44)	\	\
Type B1	24 (35)	\	\	15 (44)	\	\
Type B2	\	27 (50)	\	\	13 (50)	\
Type B3	\	13 (24)	\	\	7 (27)	\
Thymic carcinoma	\	14 (26)	\	\	6 (23)	\
Morphological features						
Diameter (cm)	4.6 ± 3.2	5.1 ± 2.2	0.330	4.4 ± 2.7	4.9 ± 1.6	0.406
Shape (round/irregular)	47/21	20/34	0.000*	22/12	8/18	0.009*
Contour (smooth/no smooth)	42/26	22/32	0.021*	24/10	10/16	0.013*
Density (uniform/non-uniform)	33/35	29/25	0.570	18/16	10/16	0.265
Calcification (yes/no)	10/58	7/47	0.782	5/29	5/26	0.874
Fat infiltration (yes/no)	22/46	37/18	0.000*	12/22	18/8	0.009*
Macrovascular involvement (yes/no)	6/62	15/39	0.006*	6/28	13/13	0.017*
Pericardial involvement (yes/no)	17/51	27/27	0.004*	8/26	16/10	0.003*

* *p* < 0.05 indicates significant difference

### Selection of radiomics features

For NE-CT, 658 features with ICC > 0.75 were retained after the consistency test, 107 features with *t*-test *p* < 0.05 were selected following the correlation test, and four features were ultimately selected after the importance test: wavelet-LLH first-order Mean, wavelet-LHL first-order Median, wavelet-LHH first-order Skewness, and wavelet-HLL glcm Idmn (Supplementary Fig. 1a, b). The NE-CT Rad-score was calculated based on these four features (Supplementary Note).

For CE-CT, 618 features with ICC > 0.75 were selected after the consistency test, 98 features with *t*-test *p* < 0.05 were selected after the correlation test, and five features were selected in the importance test: glszm SmallAreaHighGrayLevelEmphasis, wavelet-LHL first-order Median, wavelet-HLL gldm LowGrayLevelEmphasis, wavelet-HLL glrlm ShortRunLowGrayLevelEmphasis, and wavelet-HLL glszm GrayLevelNonUniformityNormalized (Supplementary Fig. 1c, d). The CE-CT Rad-score was calculated based on these five features (Supplementary Note).

### Model development and evaluation

**Fig. 3:**
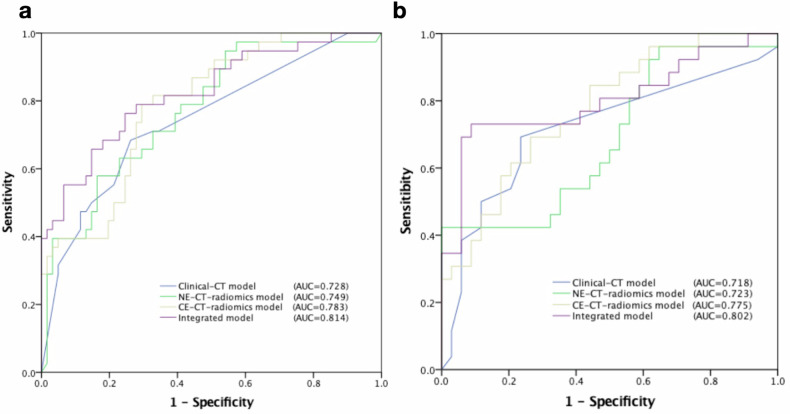
Receiver operating characteristic curves (ROCs). **a** ROCs for the clinical-CT model, non-contrast-enhanced CT (NE-CT) radiomics model, contrast-enhanced CT (CE-CT) radiomics model, and integrated model in the training cohort. **b** ROCs for the models in the external validation cohort

Multivariate logistic regression identified five morphological features (shape, contour, fatty infiltration, macrovascular, and pericardium involvement) as significantly different between low-risk and high-risk groups, while no clinical risk factors showed statistical significance. Therefore, the clinical-CT model was established using these five morphological features. As previously described, the NE-CT radiomics model and CE-CT radiomics model corresponded to the NE-CT Rad-score and CE-CT Rad-score, respectively. The AUCs for the clinical-CT model, NE-CT radiomics model, and CE-CT radiomics model in the training cohort were 0.728, 0.749, and 0.783 (Table 2, Fig. 3a), and in the external validation cohort, 0.718, 0.723, and 0.775, respectively (Table 2, Fig. 3b). The CE-CT radiomics model demonstrated better performance than the NE-CT radiomics model (*p* = 0.361), leading to the development of integrated model incorporating the CE-CT Rad-score. As shown in Table 2 and Fig. 3, the AUC of the integrated model (training cohort, 0.814; external validation cohort, 0.802) was higher than those of the other models in both training cohort (Clinical-CT model, *p* = 0.075; NE-CT radiomics model, *p* = 0.398; CE-CT radiomics model, *p* = 0.534) and external validation cohort (Clinical-CT model, *p* = 0.158; NE-CT radiomics model, *p* = 0.239; CE-CT radiomics model, *p* = 0.697).

**Table 2:** Performance of the classification of TETs predicted by different models in the training cohort and external validation cohort

AUC	Training cohort (95% CI)	External validation cohort (95% CI)
Clinical-CT	0.728 (0.646–0.811)	0.718 (0.578–0.857)
NE-CT radiomics	0.749 (0.673–0.826)	0.723 (0.592–0.854)
CE-CT radiomics	0.783 (0.713–0.853)	0.775 (0.658–0.891)
Integrated	0.814 (0.744–0.884)	0.802 (0.680–0.924)

*TETs* thymic epithelial tumors, *AUC* area under the curve, *CI* confidence interval, *NE-CT* non-contrast-enhanced CT, *CE-CT* contrast-enhanced CT

**Fig. 4:**
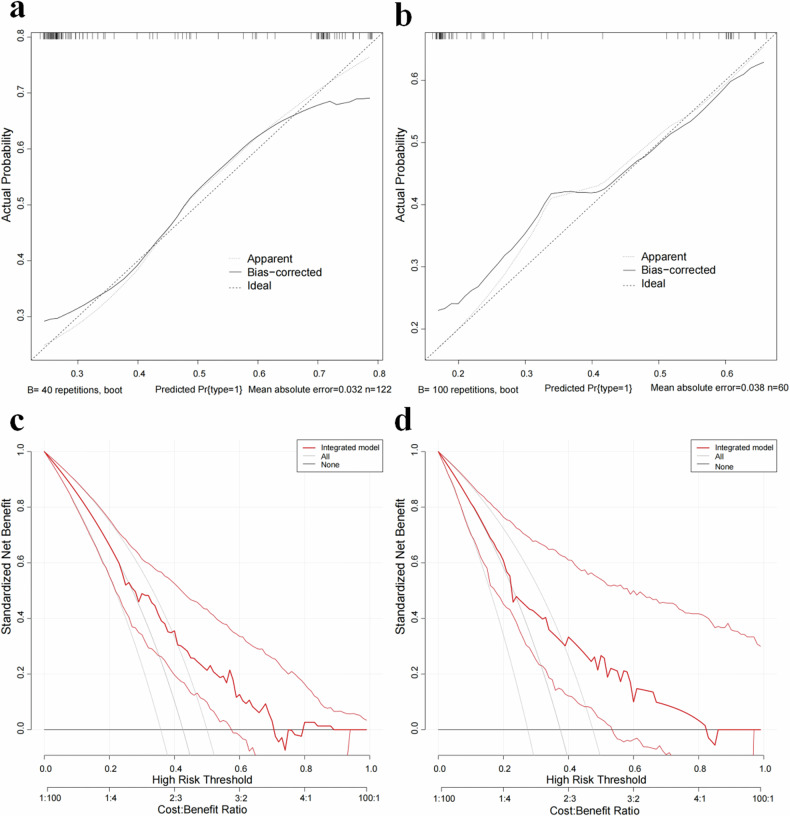
Calibration curves and decision curve analysis (DCA) for the integrated model. **a** Calibration curve for the training cohort, and (**b**) calibration curve for the external validation cohort, showing predicted probability compared to actual probability for the high-risk group. **c** DCA of the integrated model for predicting high-risk in the training cohort, and (**d**) in the external validation cohort. In the DCA, the black line represents the assumption that no patient is high-risk, the gray line represents the assumption that all patients are high-risk, and the red line represents the use of the integrated model to predict high-risk groups. The central red line in the DCA demonstrates that if the threshold probability is > 30%, the integrated model adds more net benefit than predicting all or no patients as high-risk. The lines on either side of the central red or gray line represent the 95% confidence intervals

**Fig. 5:**
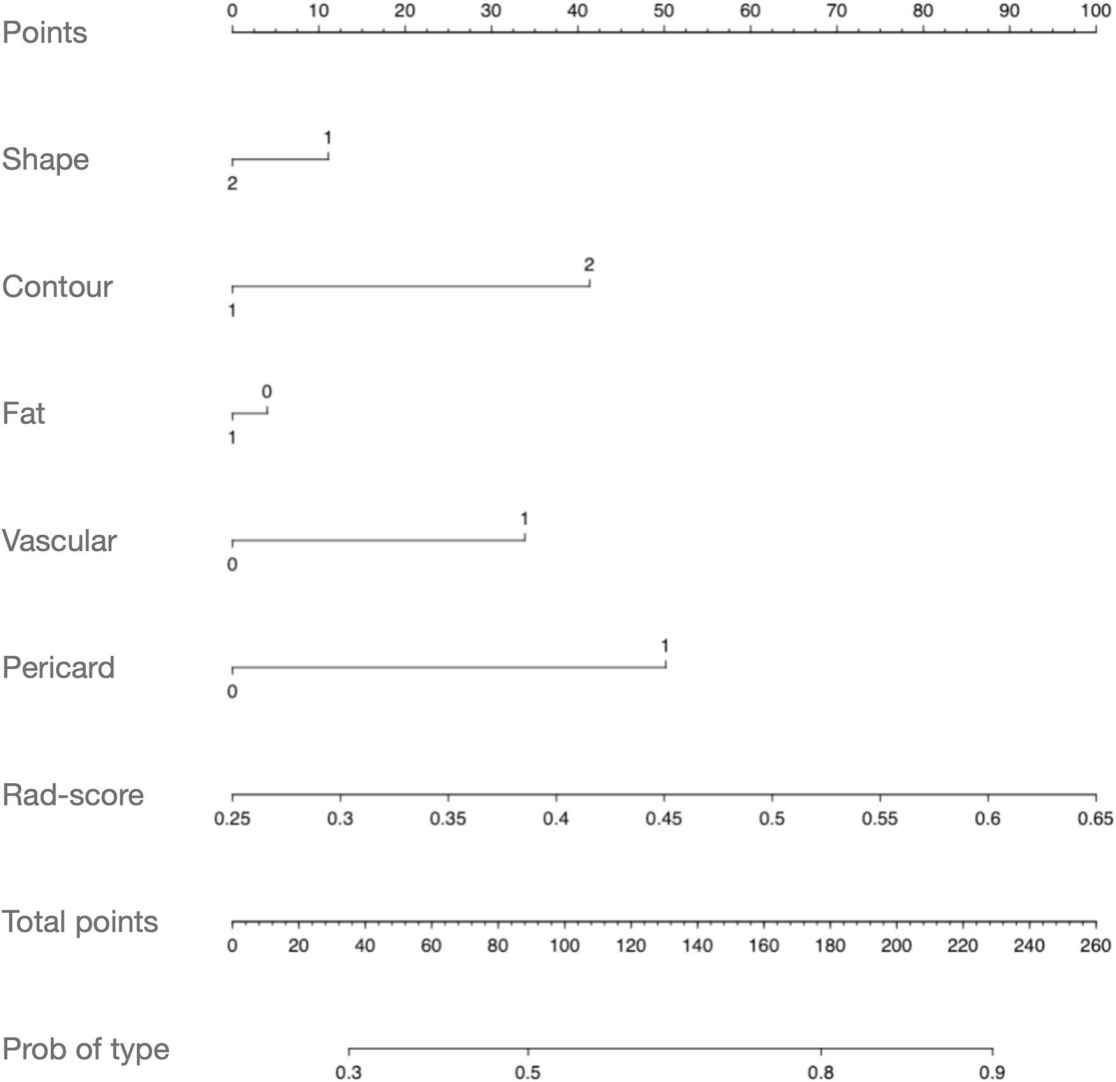
Nomogram was built in the training cohort with the shape, contour, fat, vascular, pericard, and optimal Rad-score for predicting the histological classifications of thymic epithelial tumor patients

Calibration curves revealed strong diagnostic consistency between observed outcomes and predicted high-risk group for TETs in the integrated model, both in the training cohort (Fig. 4a) and the external validation cohort (Fig. 4b). The integrated model demonstrated satisfactory alignment between the predicted probability and actual occurrence probability in identifying high-risk patients. Additionally, DCA in the training cohort confirmed the integrated model’s universal clinical practicality in distinguishing between low-risk and high-risk groups, showing a higher overall net benefit, which underscores its practical application and potential for providing additional clinical value (Fig. 4c). Similar results were observed in the external validation cohort (Fig. 4d). Figure 5 presents a nomogram that visualizes the integrated model.

## Discussion

Patients with TET in the high-risk group require multimodal treatment, including surgery, preoperative chemotherapy, and radiation therapy, which are the standard therapeutic options in several tumor centers. The selection and scope of open or minimally invasive surgery are also influenced by histological subtypes [18, 19]. Therefore, pre-treatment prediction of TET pathological types is crucial for clinical decision-making.

In this study, we developed and externally validated an integrated model using 3D NE-CT and CE-CT images to investigate the potential of radiomics in differentiating histological classifications of TET patients. Results demonstrated that the CE-CT radiomics model exhibited superior predictive capabilities compared to the NE-CT radiomics model, aligning with previous studies [20, 21] that found radiomics features from NE-CT and CE-CT to be effective non-invasive biomarkers for distinguishing between low-risk and high-risk TETs, with CE-CT performing slightly better. Notably, artifacts of the lesions in some NE-CT images can affect the reliability of tumor heterogeneity assessments, and unclear edges can complicate lesion segmentation. Our study utilized venous phase images for image segmentation in the enhanced sequence, enhancing lesion visibility and segmentation accuracy. Radiomics features from CE-CT may reflect the heterogeneity through blood supply and contrast distribution differences across intravascular, extravascular, and extracellular spaces [22], allowing CE-CT radiomics features to reflect tumor heterogeneity more effectively. However, most studies have been limited to single NE-CT or CE-CT, lacking a comprehensive evaluation of the impact of contrast agents on radiomics [12–16]. In this study, we found that the most robust radiomics feature is wavelet-LHL first-order Median, which was selected into both NE-CT Rad-score and CE-CE Rad-score. This may be attributed to the fact that the CT images can demonstrate the vascular enhancement characteristics of TET, and the wavelet-LHL first-order Median is more sensitive to the image outline. Consequently, the feature can more effectively reflect the information about the neurovascular components and heterogeneity in TET. In addition, the 3D segmentation for radiomics analysis employed in our study is another advantage, which enhances the precision of radiomics-driven predictions by providing a more comprehensive image information.

Radiomics features demonstrated favorable efficacy in distinguishing between low-risk and high-risk TETs. However, it should be noted that radiomics is not the sole non-invasive approach for predicting the classification of TETs. Clinical risk factors and morphological features have also been employed to assess the risk of TETs as they can be easily obtained and are not influenced by variations in CT sequences. In line with prior studies [23–27], irregular shape, non-smooth contour, fatty infiltration, macrovascular involvement, and pericardium involvement are associated with a higher risk of malignancy in TET patients. This substantiates the potential use of CT morphological features for predicting TET classification, though radiomics features generally offer superior performance, as they more effectively reflect tumor heterogeneity compared to CT morphological features [12–16]. Consequently, combining radiomics and morphological features may improve the accuracy of distinguishing high-risk from low-risk TETs. For this objective, an integrated model incorporating morphological features and CE-CT radiomics features was developed. This integrated model exhibited the highest predictive capability and effectively distinguished different subtypes of TETs, consistent with previous studies [13–16, 20, 21]. These findings indicate that the developed integrated model, combining CE-CT radiomics features and morphological features, could serve as a non-invasive tool to distinguish between low-risk and high-risk groups, supporting better-informed treatment decisions. However, the current investigation reveals that the AUC of integrated model was somewhat lower than those reported in previous studies, potentially due to differences in sample size or the utilization of examination tools.

There are several limitations in this study. First, it is a retrospective study that inherently carries selection biases and is based on a relatively small sample size. Although datasets were independently collected from two hospitals, larger prospective multicenter studies are necessary to validate these findings. Second, the radiomics features derived from different CT equipment may influence the results. However, the image acquisition parameters remain consistent to alleviate this difference. Third, manual segmentation of lesions may affect the observer subjective. To address this potential bias, two radiologists with over a decade of experience in chest radiology independently delineated the tumors, and the ICC was also assessed. Furthermore, it is imperative to undertake further research and develop advanced auto-segmentation techniques to reduce subjective segmentation variability. Finally, radiomics features derived from magnetic resonance imaging [28, 29] have shown greater accuracy than CE-CT in predicting TET histological classifications. Further studies will incorporate multimodal data to provide additional useful information for identifying lesions in subsequent endeavors.

In conclusion, optimized radiomics features from CE-CT exhibited superior predictive performance over NE-CT, and an integrated model established by CE-CT radiomics features and morphological features may aid in identifying histological classifications of thymic epithelial tumors.

## Supplementary information

ELECTRONIC SUPPLEMENTARY MATERIAL

## Data Availability

The data used or analyzed during the current study are available from the corresponding author upon reasonable request.

## Abbreviations

AUC: Area under the curve
CE-CT: Contrast-enhanced computed tomography
DCA: Decision curve analysis
GLCM: Gray level co-occurrence matrix
GLDM: Gray level dependence matrix
GLRLM: Gray level run length matrix
GLSZM: Gray level size zone matrix
ICC: Intraclass correlation coefficient
LASSO: Least absolute shrinkage and selection operator
NE-CT: Non-contrast-enhanced computed tomography
Rad-score: Radiomics score
ROC: Receiver operating characteristic
TET: Thymic epithelial tumor
VOI: Volume of interest
