# Supplementary material for: Using optimized CT type to predict histological classifications of thymic epithelial tumors: a radiomics integrated analysis
Source: Insights Imaging. 2025 Mar 22;16:67. doi: 10.1186/s13244-025-01933-7 (PMC11929666; doi:10.1186/s13244-025-01933-7)
Supplement: Supplementary file 1 — ELECTRONIC SUPPLEMENTARY MATERIAL [file 13244_2025_1933_MOESM1_ESM.pdf]

Using optimized CT type to predict histological classifications of thymic epithelial tumors: a radiomics integrated analysis

ELECTRONIC SUPPLEMENTARY MATERIAL

Supplementary Note: Rad-Score

NE-CT Rad-score = 26.984 × wavelet-HLL glcm Idmn
+ $3.868e^{-2}$ × wavelet-LHL first-order Median
+ $4.811e^{-3}$ × wavelet-LLH first-order Mean
- $3.249e^{-2}$ × wavelet-LHH first-order Skewness

CE-CT Rad-score = $5.710e^{-2}$ × wavelet-LHL first-order Median
+ $7.921e^{-5}$ × glszm_SmallAreaHighGrayLevelEmphasis
- 3.413 × wavelet-HLL glrlm_ShortRunLowGrayLevelEmphasis
- 1.696 × wavelet-HLL gldm_LowGrayLevelEmphasis
- 1.201 × wavelet-HLL glszm_GrayLevelNonUniformityNormalized

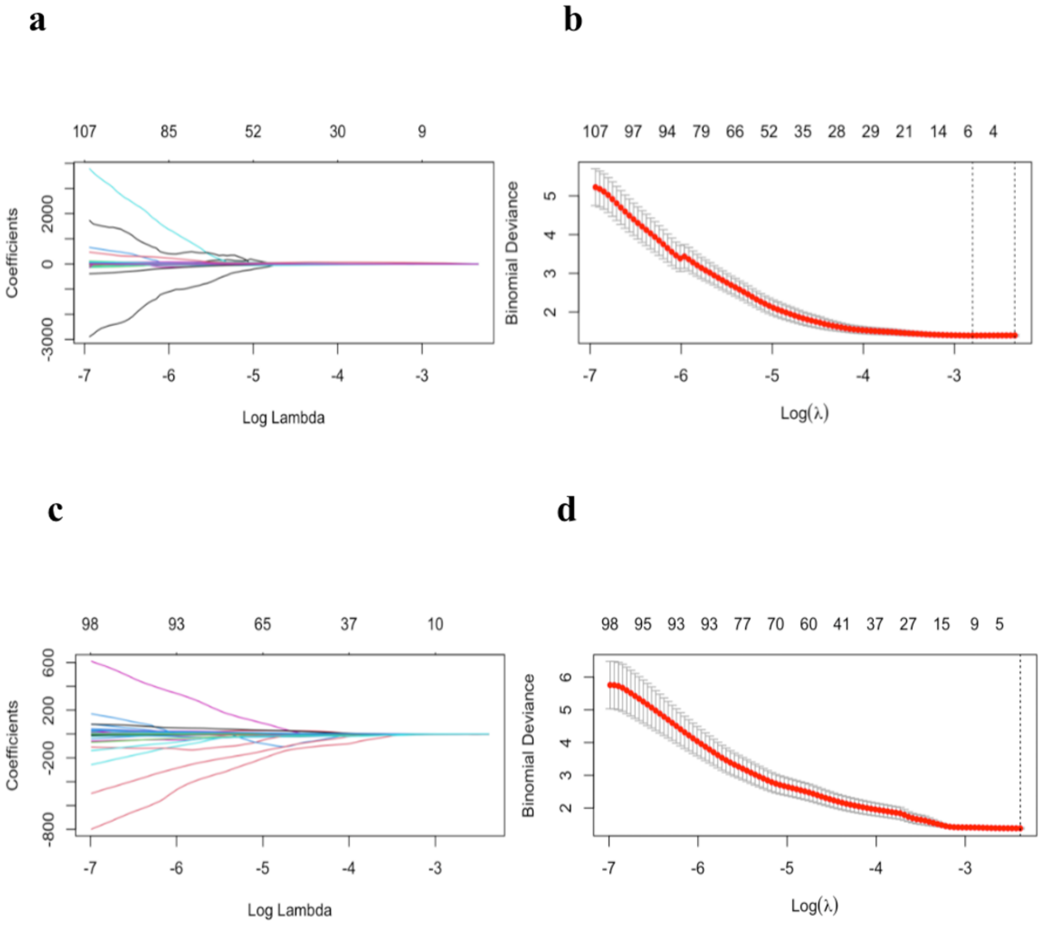

Supplementary Fig.1 Selection of radiomics features using the LASSO regression. NE-CT (a, b). CE-CT (c, d). LASSO coefficient profiles of whole features, coefficient profiles are plotted against log(λ) (a, c). The tuning parameter (λ) was selected using 10-fold cross-validation based on minimum criteria, Log(λ) is plotted on the x-axis, and binomial deviance is plotted on the y-axis (b, d). Four optimal radiomics features with non-zero coefficients were indicated in the NE-CT, and five optimal radiomics features with non-zero coefficients were indicated in the CE-CT.
